# A Fully Automated Trial Selection Method for Optimization of Motor Imagery Based Brain-Computer Interface

**DOI:** 10.1371/journal.pone.0162657

**Published:** 2016-09-15

**Authors:** Bangyan Zhou, Xiaopei Wu, Zhao Lv, Lei Zhang, Xiaojin Guo

**Affiliations:** 1 Key Laboratory of Intelligent Computing & Signal Processing, Ministry of Education, Anhui University, Hefei, China; 2 School of Computer Science and Technology, Anhui University, Hefei, China; University of Minnesota, UNITED STATES

## Abstract

Independent component analysis (ICA) as a promising spatial filtering method can separate motor-related independent components (MRICs) from the multichannel electroencephalogram (EEG) signals. However, the unpredictable burst interferences may significantly degrade the performance of ICA-based brain-computer interface (BCI) system. In this study, we proposed a new algorithm frame to address this issue by combining the single-trial-based ICA filter with zero-training classifier. We developed a two-round data selection method to identify automatically the badly corrupted EEG trials in the training set. The “high quality” training trials were utilized to optimize the ICA filter. In addition, we proposed an accuracy-matrix method to locate the artifact data segments within a single trial and investigated which types of artifacts can influence the performance of the ICA-based MIBCIs. Twenty-six EEG datasets of three-class motor imagery were used to validate the proposed methods, and the classification accuracies were compared with that obtained by frequently used common spatial pattern (CSP) spatial filtering algorithm. The experimental results demonstrated that the proposed optimizing strategy could effectively improve the stability, practicality and classification performance of ICA-based MIBCI. The study revealed that rational use of ICA method may be crucial in building a practical ICA-based MIBCI system.

## Introduction

Noninvasive brain-computer interfaces (BCIs) measure brain activities, and translate them directly into controlling commands to operate external devices without resorting to the peripheral muscular nerve system [1–3]. A common input for BCI systems is the scalp-recorded electroencephalogram (EEG) signal reflecting the electric field generated by the spontaneous electrophysiological activities of neurons. However, recorded EEGs are inevitably contaminated with non-brain activity artifacts [4, 5] such as electromyograms (EMGs), electrooculograms (EOGs), electrocardiograms (ECGs) and various environmental electromagnetic interferences. Furthermore, EEGs are also characterized by low spatial resolution due to the volume conduction effect of the human brain. Such restriction greatly deteriorates the BCI performance, which is usually addressed by the use of spatial filter techniques.

As is known that, two spatial filters namely, common spatial patterns (CSP) [6–11] and independent component analysis (ICA) [12–17] are widely used in motor imagery BCI (MIBCI) systems. CSP adopts a supervised algorithm that needs plenty of labeled data to find a projection matrix for maximizing the differences between the variances of two-class EEG data. Besides, the selected data are suggested to have strong desynchronization/synchronization (ERD/ERS) phenomena [18–19]. As such, the CSP method requires the “high quality” training data with accurate labels. These requirements can be greedy, since the distraction and fatigue of subjects in the long data collection process often produce mislabeled trials. ICA is a relatively new blind source separation (BSS) technique [12,16]. Based on the independence assumption of the signal sources, ICA can extract the hidden sources and the corresponding mixing model from a set of measured signals. Compared with the CSP algorithm, ICA is an unsupervised and model-based algorithm. Theoretically, arbitrary continuous EEG segments can be used to calculate the ICA spatial filters [15, 20], which facilitates BCI operation and relieves the pressure of BCI users in the training data collection.

However, ICA algorithm also exhibits its own problems when applied to MIBCI. The permutation problem is a frequently discussed issue [14], which remains an obstacle to the automatic selection of motor related independent components (MRICs). Usually, manual visual inspections based on topographic maps of spatial patterns or frequency spectrum features of independent components (ICs) are performed during the training phase [21–22], but these methods are time-consuming. Some automatic identification methods were developed by using the frequency-spatial features or predefined matching templates [20, 23–26]. These methods are sometimes unsatisfied to deal with the strong nonstationary and noisy EEGs. We have reported the degraded performance of ICA caused by random interferences and artifacts (such as the burst artifacts induced by involuntary body movement or electrode loosening) [27–28]. This is because the wide-band burst artifacts are hardly eliminated by temporal filters, and moreover, they usually are of short duration and irregular appearance, which are difficult for ICA to separate them into a single output channel.

In this study, we proposed a novel strategy to solve the aforementioned problems. The rationale is to recognize the badly-corrupted EEG trials, i.e., the bad trials through two rounds of EEG trials selection by combining the single-trial-based ICA filter and the zero-training classifier. We can thus obtain more accurate ICA mixing models and corresponding MRICs detection filters by rejecting the bad trials. That also guaranteed the reliability of the ICs automatic identification algorithm implemented only using the spatial pattern of ICA sources. Meanwhile, a simple and effective classification rule, based on variance comparison of MRICs within *mu/beta* rhythm frequency bands, was designed to construct the zero-training classifier. Furthermore, we employed an accuracy-matrix based visualization technique by using consecutively overlapping EEG segments from each trial to calculate ICA filters. The purpose was to further locate the artifact data segments within a single trial, so that we can assess the influences of different types of artifacts on the performance of ICA-MIBCI system.

This paper is organized as follows: Section 1, introduces the detailed experimental paradigm of the EEG data collection, and explains the ICA and CSP algorithms. Section 2, explains the algorithm frame of ICA-based MIBCI, showing how “bad trials” can be recognized by two-round data selection, and how to optimize ICA filter calculation. Section 3 compares the experimental results of the three methods (ICA, optimized ICA and CSP). Section 4, investigates the effects of different artifacts on ICA-based MIBCI performance with a proposed technique of accuracy-matrix based visualization. The last section concludes the paper and proposes suggestions for future work.

## Methods

### 2.1 Experiment paradigm and EEG recording

Four healthy subjects (one male and three females, aged between 22 and 28) participated in the motor imagery experiment. All subjects are graduate students in our laboratory, with prior experience in the experimental paradigm. The study was approved by the Institutional Review Board at Anhui University. Written informed consent has been obtained from each subject.

**Fig 1:**
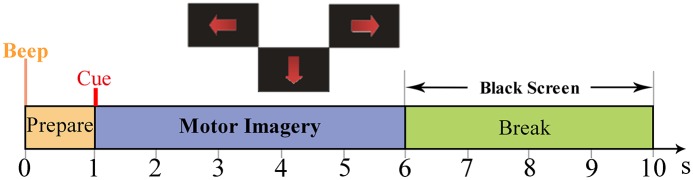
Timing scheme of paradigm.

Throughout the experiment, the subject sat in a comfortable armchair facing a computer screen. The duration of each trial was 10 s as shown in Fig 1. A trial started by a short beep indicating 1 s preparation time, and followed by a red arrow pointing randomly to three directions (left, right, or bottom) lasting for 5 s and then presented a black screen for 4 s. The subject was instructed to immediately perform the imagination tasks of the left hand, right hand or foot movement respectively according to the cue direction, and try to relax during the black screen.

**Fig 2:**
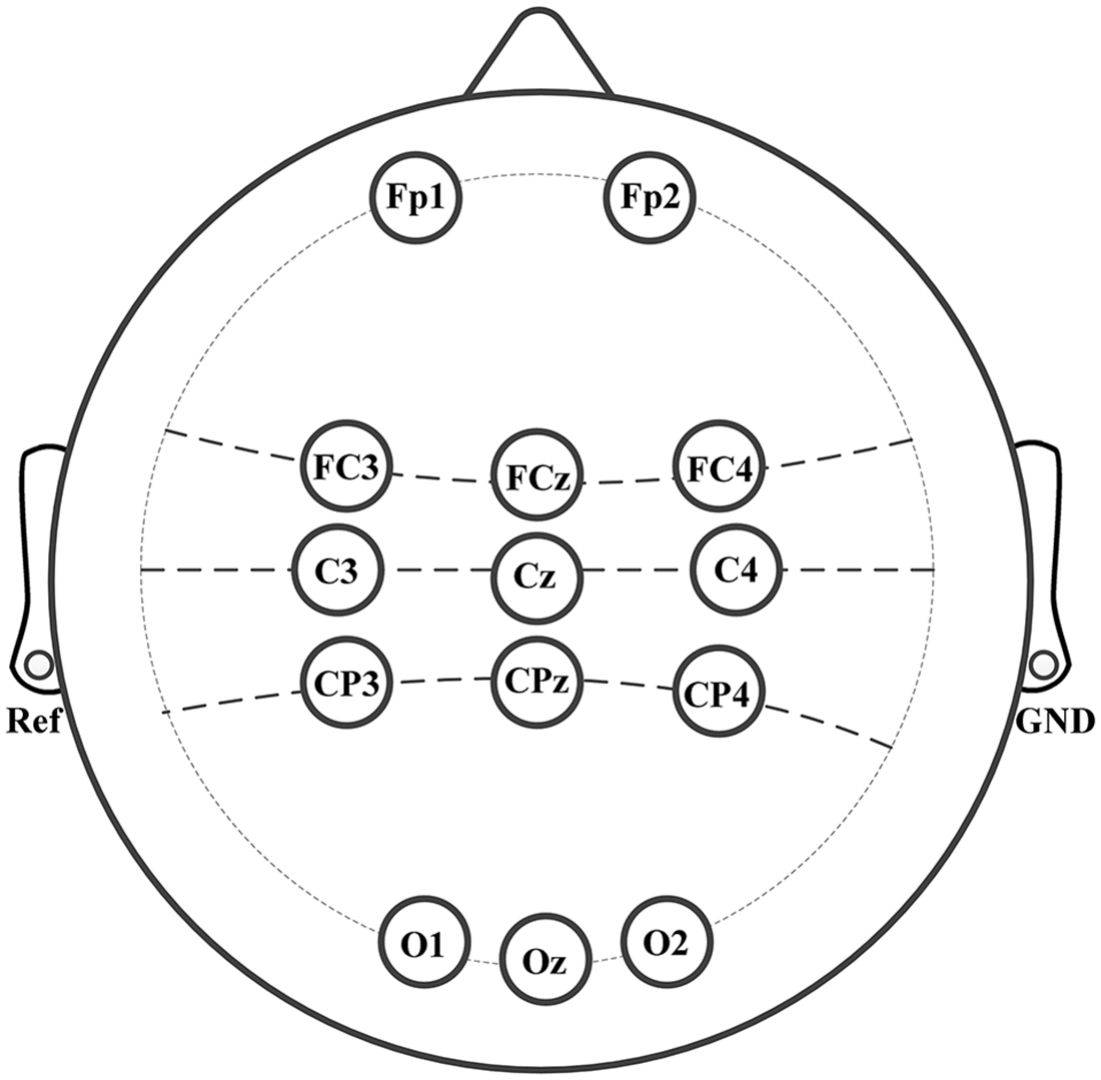
Layout of EEG electrodes with standard international 10–20 system.

EEG data were recorded from 14 scalp electrodes placed at locations according to the standard international 10–20 system (Fig 2), with the left mastoid served as the reference and the right mastoid as the ground. The raw EEGs were band-pass filtered between 0.1 and 100 Hz, and digitally sampled at 250 Hz. An additional 50 Hz notch filter was applied to suppress the power line interference. Every subject went through three sessions, each of which contained two consecutive runs with several minutes inter-run breaks, and each run comprised 75 trials (25 trials per class). The intervals between two sessions varied from several days to several months.

Prior to the calculation of ICA spatial filters, the recorded EEG data were filtered with a zero-phase FIR band-pass filter between 8–30 Hz, which covers the motor-related *mu* and *beta* rhythm. We analyzed the time frequency maps of three electrode (C3, Cz, C4) signals [29]. The testing frequency bands with obvious band power fluctuation during three-class motor imagery tasks were selected. The most active frequency bands were 10–14 Hz for three subjects (S1, S2 and S4), 12–16 Hz for subject S3. The time segment of 0.5–5 s of a trial was chosen for accuracy testing and also for CSP filter calculation. Two different electrode-distributions were defined, one is an eight-channel scheme (FP1, FP2, C3, Cz, C4, O1, Oz, O2) and the other is a nine-channel scheme (FC3, FCz, FC4, C3, Cz, C4, CP3, CPz, CP4). We chose the one with higher classification accuracy for a subject based on the previous analysis.

### 2.2CSP algorithm

CSP is a proven method for effective spatial filtering in MIBCI [6–9]. Assuming **X**_1_ and **X**_2_ are *N*-channel EEG data of two-class motor imagery. All channel data are centered and scaled. Let **Ʃ**^(+)^ and **Ʃ**^(-)^ represent the variances of two-class motor imagery EEG data within specific time segment, which are estimated as follows.

$$\Sigma^{\left(+\right)}=E(X_{1}X_{1}^{Τ})\;;\;\Sigma^{\left(-\right)}=E(X_{2}X_{2}^{Τ})$$ (1)

Since the covariance matrices **Ʃ**^(+)^ and **Ʃ**^(-)^ are real symmetric matrix, there is an orthogonal matrix **W**, which can project the variance matrix into a diagonal matrix.

$$\begin{array}{l} W^{Τ}\Sigma^{\left(+\right)}W=\Lambda =diag(\lambda_{1},\cdots ,\lambda_{N})\\ W^{Τ}\Sigma^{\left(-\right)}W=I-\Lambda \end{array}$$ (2)

where **I** is an *N*×*N* identity matrix, *λ*_*i*_ (*i* = 1,…,*N*) is the eigenvalue of **Ʃ**^(+)^. In this study, one pair of eigenvectors corresponding to the maximum and minimum eigenvalues were used to construct the CSP spatial filters.

For three-class motor imagery problem, we employed the one-versus-the-rest (OVR) [10–11] method by dividing the multi-class problem into several binary decisions. In this study, six groups of two-class CSP filters were calculated to obtain three predicted results, and the final class label was chosen by the voting algorithm.

### 2.3 ICA algorithm

The basic ICA model assumes that the measured *N*-channel EEG data **x**(*t*) = [***x***_1_(*t*),…,***x***_*N*_(*t*)]^T^ are noiseless linear and instantaneous mixtures of several latent independent sources **s**(*t*) = [***s***_1_(*t*),…,***s***_*N*_(*t*)]^T^.

x(t)=As(t) (3)

where **A** = [***a***_1_,…,***a***_*N*_] is a mixing matrix; The column vector ***a***_*i*_(*i* = 1,2,..,*N*) is called a spatial pattern, which reflects the weights of the independent source ***s***_*i*_(*t*) (*i* = 1,2,..,*N*) projecting to the measured EEG signals **x**(*t*). The goal of different ICA algorithm is to estimate the hidden independent sources with a separating matrix **W = [*w***_1_,…,***w***_*N*_], i.e.,

u(t)=Wx(t) (4)

where **u(***t***)** = [***u***_1_(*t*),…,***u***_*N*_(*t*)]^T^ is the estimated independent sources. Under the premise that the sources **s**(*t*) and mixing matrix **A** are both unknown, the key assumption used to recover the source signals is the statistical independence of the sources. The ICA algorithm based on the principle of information maximization [30] was used in this work, and the object function was optimized by the natural gradient algorithm [31]. The separating matrix **W** was initialized as a diagonal matrix with same positive main diagonal entries and the final iterative formula is as follows:

$$\Delta W\propto [I-E[Ktanh(u)u^{T}+uu^{{}_{T}}]]W$$ (5)

where *E*[·] is the statistical average. **K** is a *N*×*N* switch matrix with diagonal elements *k*_*ii*_ equals 1 or -1 corresponding to super-Gaussian or sub-Gaussian sources respectively, which can be estimated by the signs of Kurtosis of **u**(*t*). The variance of sources **u**(*t*) was normalized to eliminate the influence of the amplitude uncertainty of the recovered sources, and the elements of mixing matrix **A** and separating matrix **W** are adjusted accordingly as follows:

$$\begin{array}{l} u(t)\leftarrow u(t)/diag[std(u(t))]\\ A\leftarrow Adiag[std(u(t))],W=A^{-1} \end{array}$$ (6)

where *std*(·) is the standard deviation vector; *diag*(·) transforms the vector to the diagonal matrix. In this work, we wrote the ICA codes by ourselves instead of using the standard Infomax algorithm in EEGLAB [32].

## The Algorithm Frame of ICA-Based MIBCI

**Fig 3:**
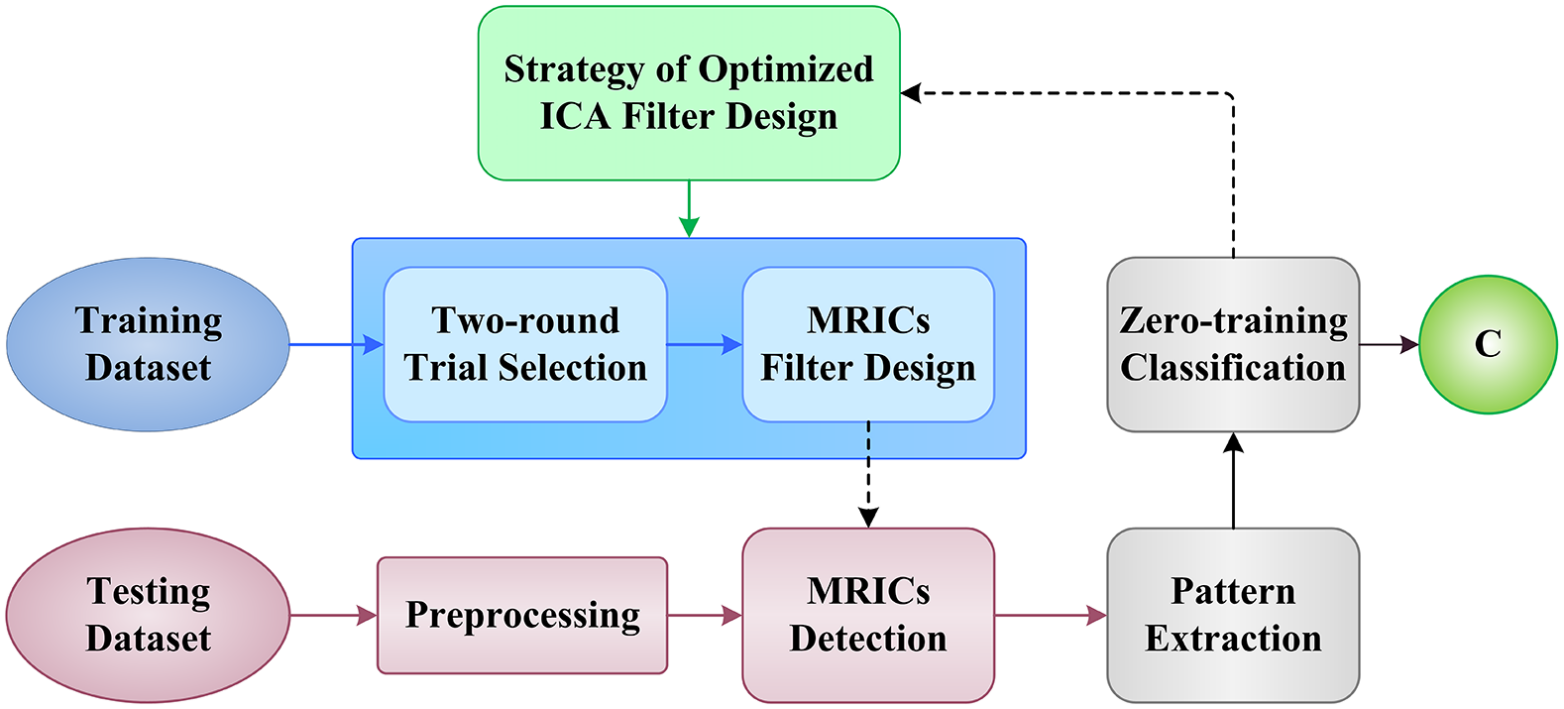
Illustration of proposed algorithm frame for ICA-based BCI system.

The structure of our algorithm frame is illustrated in Fig 3. For the purpose of ICA filter optimization and reliable MRICs extraction, we designed a two-round trial selection strategy to identify the “good trials” and “bad trials” in training sets. The following sections will describe the details of our proposed methods.

### 3.1 MRICs and its detection filters

In the training phase, after applying ICA to the EEG segments, we need to identify the MRICs based on spatial or frequency features, and then select corresponding rows of separating matrix **W** to be the spatial filters for detecting MRICs in the testing phase. Based on the physiological knowledge, the limb movement imagination can induce the ERD phenomenon [18–19], i.e., left/right hand motor imagery will cause mu/beta-band power depression at contralateral primary motor cortex (C4/C3), and foot movement imagination ERD occurs near the middle area of primary motor cortex (Cz). Therefore, corresponding to the three electrodes, we selected three MRICs, whose features were used for classifying the three-class motor imageries.

Previous studies [33–34] revealed that each task-related brain sources has unique and consistent scalp maps in similar EEG experimental paradigms. Each column of ICA mixing matrix **A** represents the projections of ICs on the scalp electrodes. We only employed the spatial patterns of ICs for the selection of MRICs and its detection filters. However, ICA models can be accurately estimated in any case. The reason is that scalp EEG signals used to calculate ICA models are inevitably contaminated with different types of artifacts, and that may cause inaccurate ICA calculation as well as the wrong choice of MRICs and their detection filters. In section 5.1, we will show that if the badly-corrupted EEG trials are used for ICA learning, the performance of MRICs detection filters may be seriously degraded. For these reasons, this study focuses on trial-selection-based ICA filter optimization instead of using complicated MRICs auto selection algorithm.

### 3.2 Trial analysis and selection

**Fig 4:**
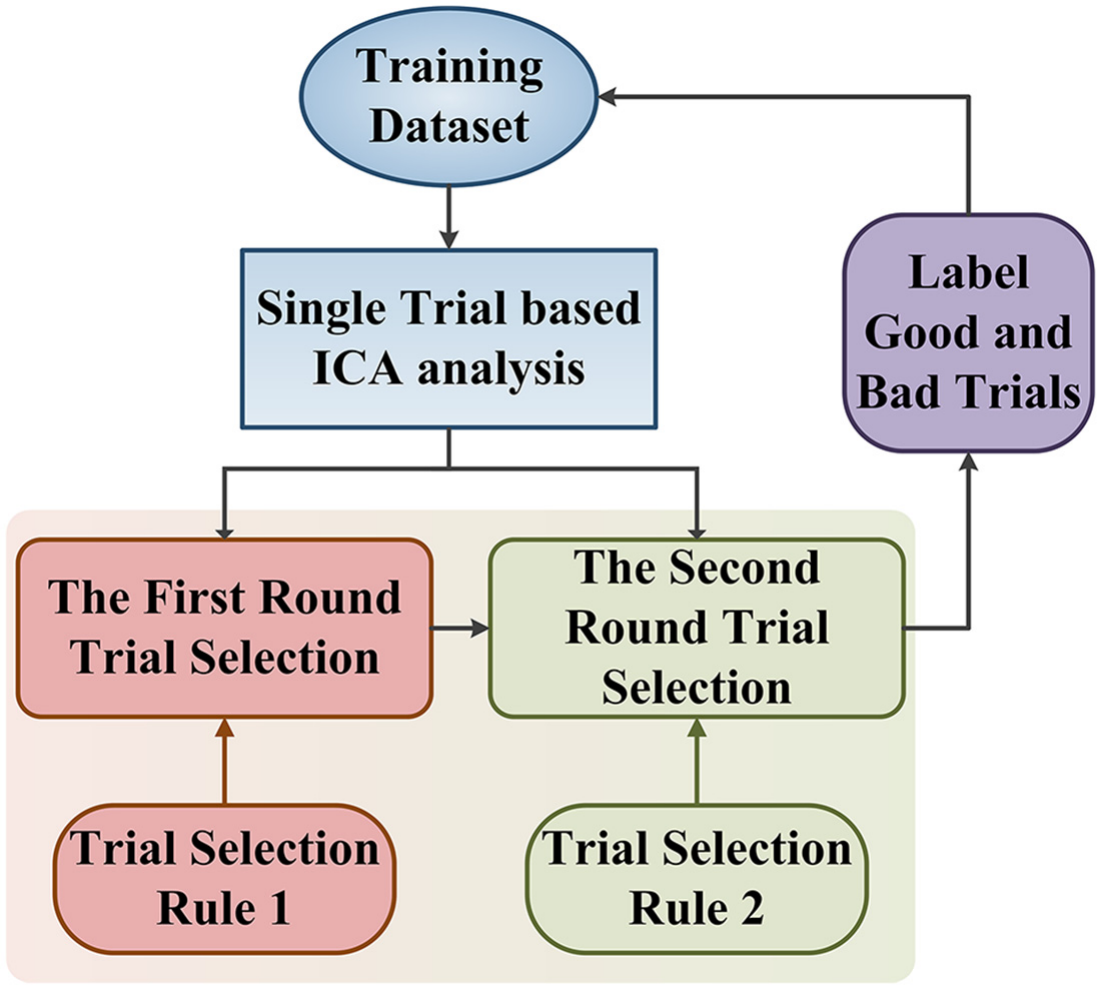
The block diagram of two-round trial selection method.

The two-round trial selection method is designed for optimizing ICA filters and illustrated in Fig 4.

#### 3.2.1 The first-round trial selection based on spatial patterns of ICs

In the first round trial selection, a single-trial based ICA analysis was performed on labeled training dataset, which contained *I* EEG trials {x_*i*_, *i =* 1,…,*I*} of three-class motor imagery. It will then yield mixing/separating matrix pairs {A_*i*_, W_*i*_, *i =* 1,…,*I*} and *I* groups of ICs {u_*i*_, *i =* 1,…,*I*}.

**Fig 5:**
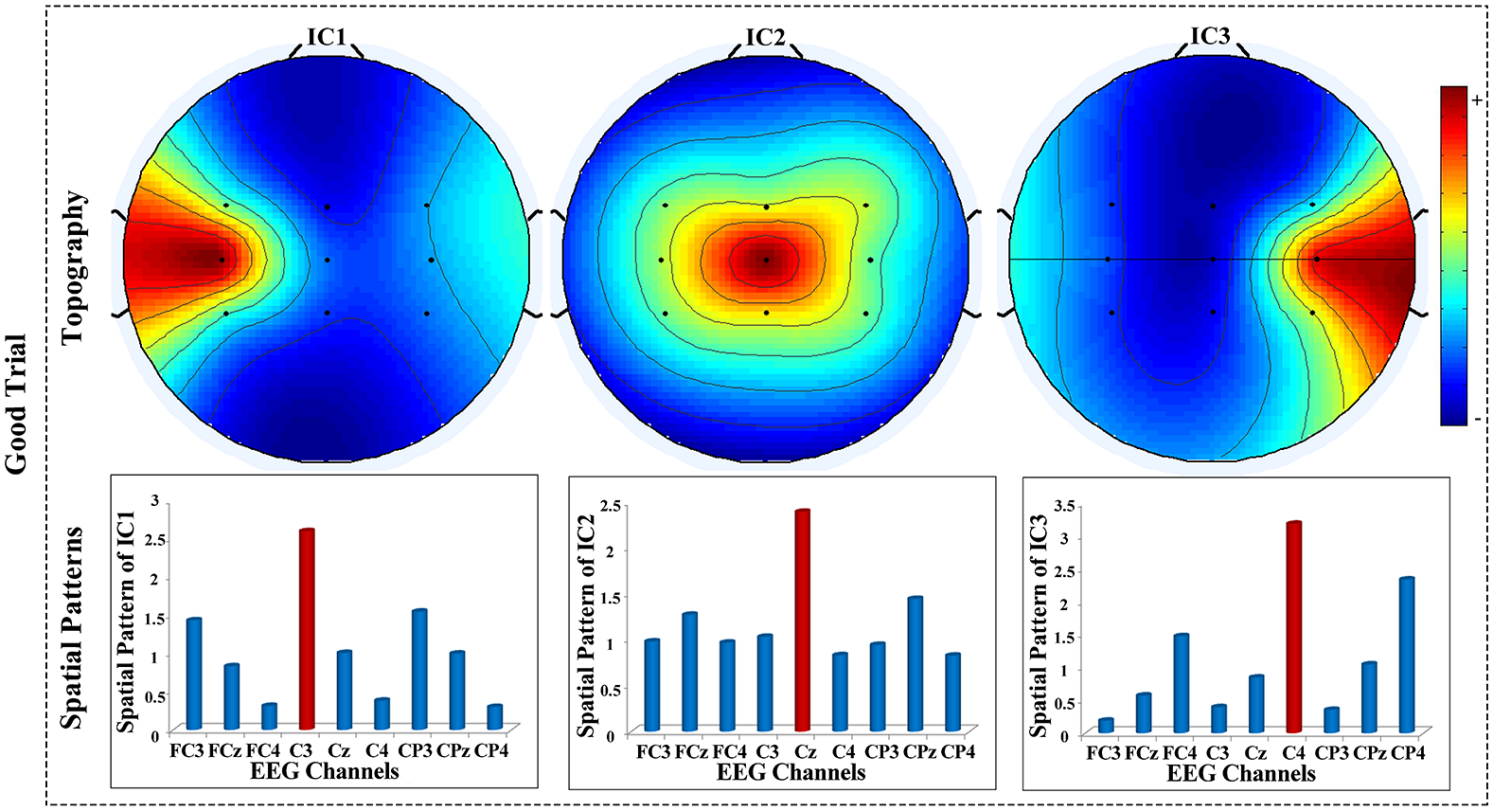
Templates of MRICs topography maps and spatial patterns of a “good trial”.

**Fig 6:**
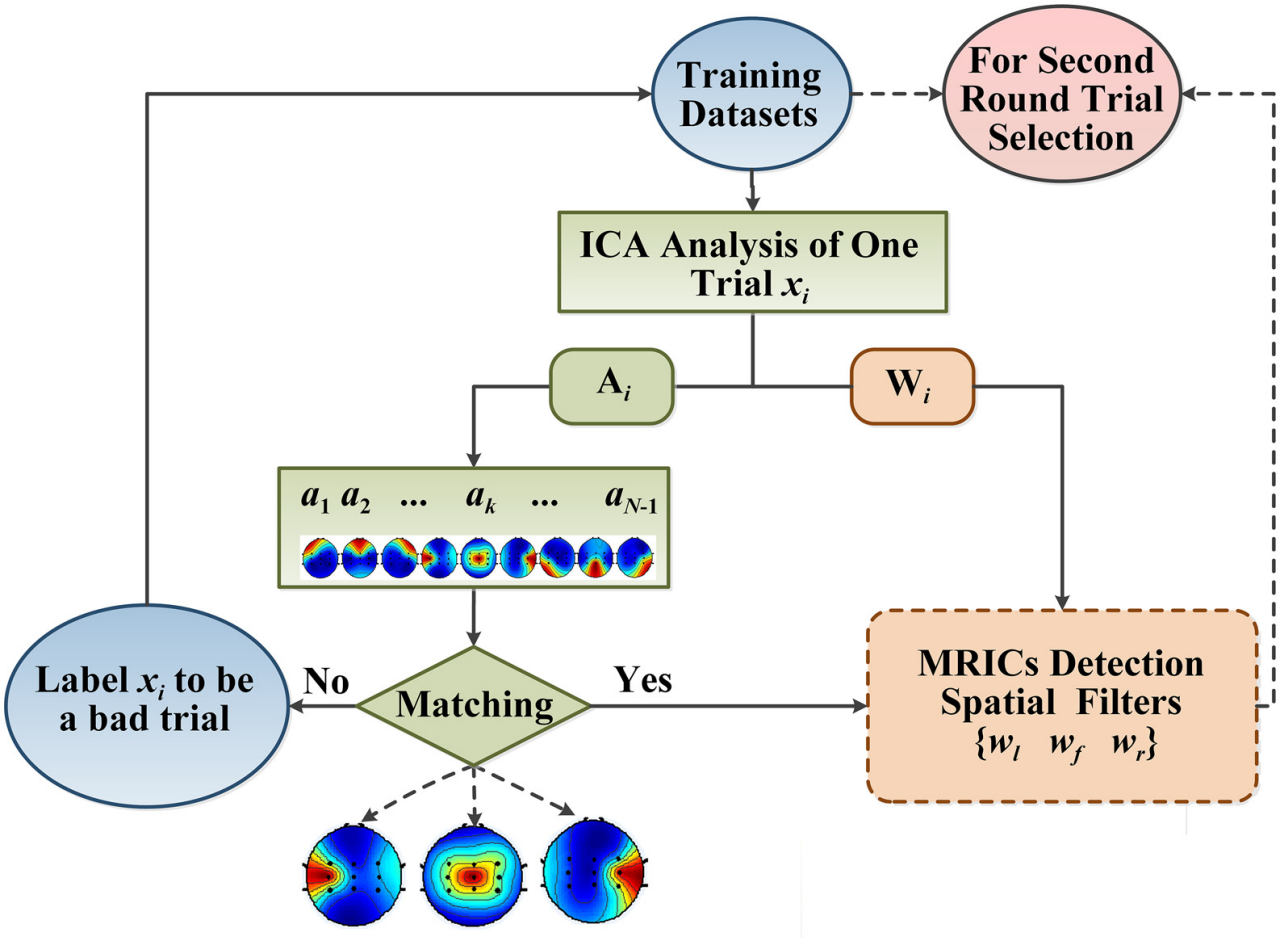
The schematic drawing of the first-round trial selection algorithm.

The criterion for the first-round trial selection is that **A**_*i*_ of a candidate EEG trial must simultaneously contain three columns, i.e., the projection maps of three ICs, being similar to the templates of MRICs topography maps in Fig 5. If **A**_*i*_ of a single-trial ICA satisfies this criterion, the *i*th trial will be labeled as a “good trial”, otherwise, it will be labeled as a “bad trial”. The details of first-round trial selection are described in Fig 6.

Since motor-related brain sources should have the maximum projection weights on the nearest detection electrodes, the "Matching" step in Fig 6 is realized by inspecting and comparing the entries along each column of mixing matrix **A**_*i*_ trying to find three ICs with maximum projections on C3, Cz and C4 positions respectively. Failed to find these ICs, this trial was labeled as “bad trial”. If there were multiple ICs with a maximum projection on one of the three specified channels, the one with the highest projection weights was selected.

**Fig 7:**
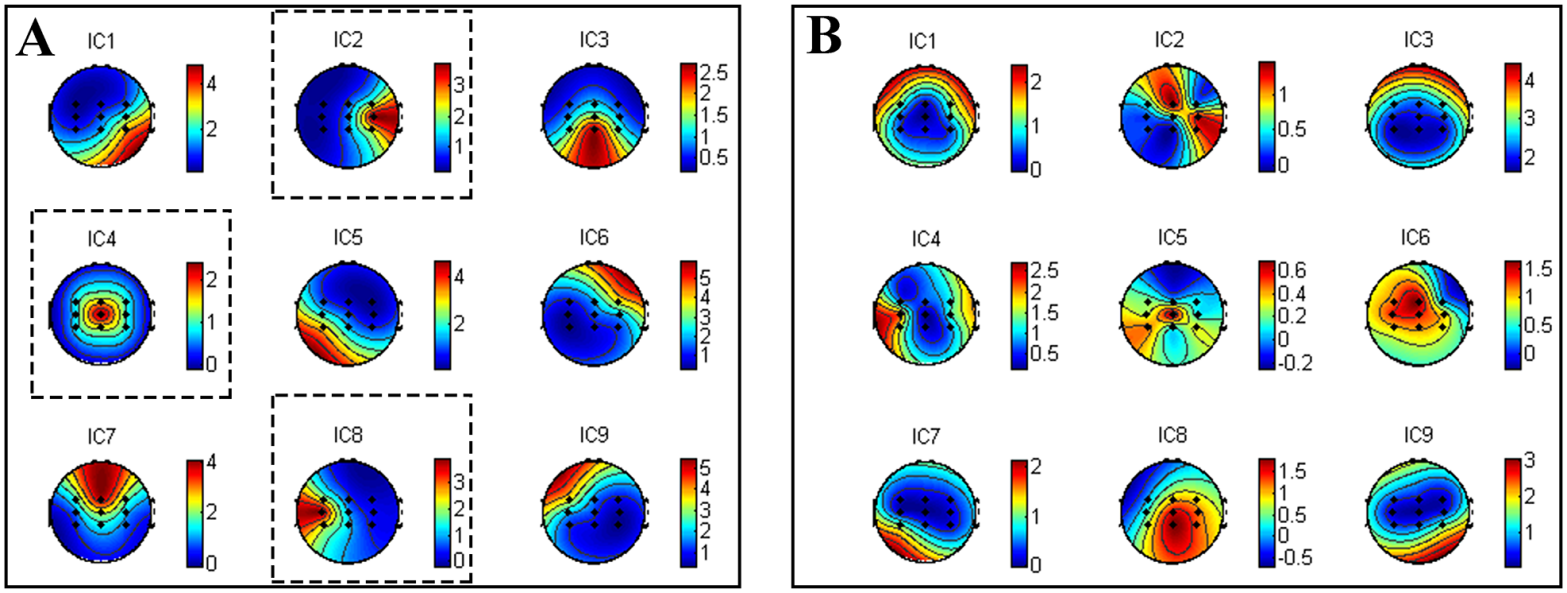
The topography maps of nine ICs calculated on two trials by ICA algorithm. (A) “good trial” (B) “bad trial”.

In Fig 7, we illustrate two groups of scalp maps of ICs related to a good trial (Fig 7A) and a bad trial (Fig 7B). In this case, the channels of vector **x**_*i*_ are FC3, FCz, FC4, C3, Cz, C4, CP3, CPz, CP4. We can find 3 maps (IC2, IC4 and IC8 in Fig 7A) well-matched with Fig 5, while that is not the case for Fig 7B. Thus the EEG trial corresponding to Fig 7A should be labeled as "good trial" in the training dataset. And, the corresponding MRICs detection filters ***w***_*l*_, ***w***_*r*_, ***w***_*f*_ for left hand, right hand and foot movement imagination were also saved in the filter-datasets for the second-round trial analysis and selection. After the first-round trial selection step, we may assume to have *P* good trials: {**x**_*j*_, *j =* 1,…,*P*≤*I*} with the corresponding spatial filter sets: {**w**_*j*_ = [***w***_*l*_, ***w***_*f*_, ***w***_*r*_], *j =* 1,…,*P*} in the training dataset.

#### 3.2.2 The second-round trial selection based on classification accuracies

**Fig 8:**
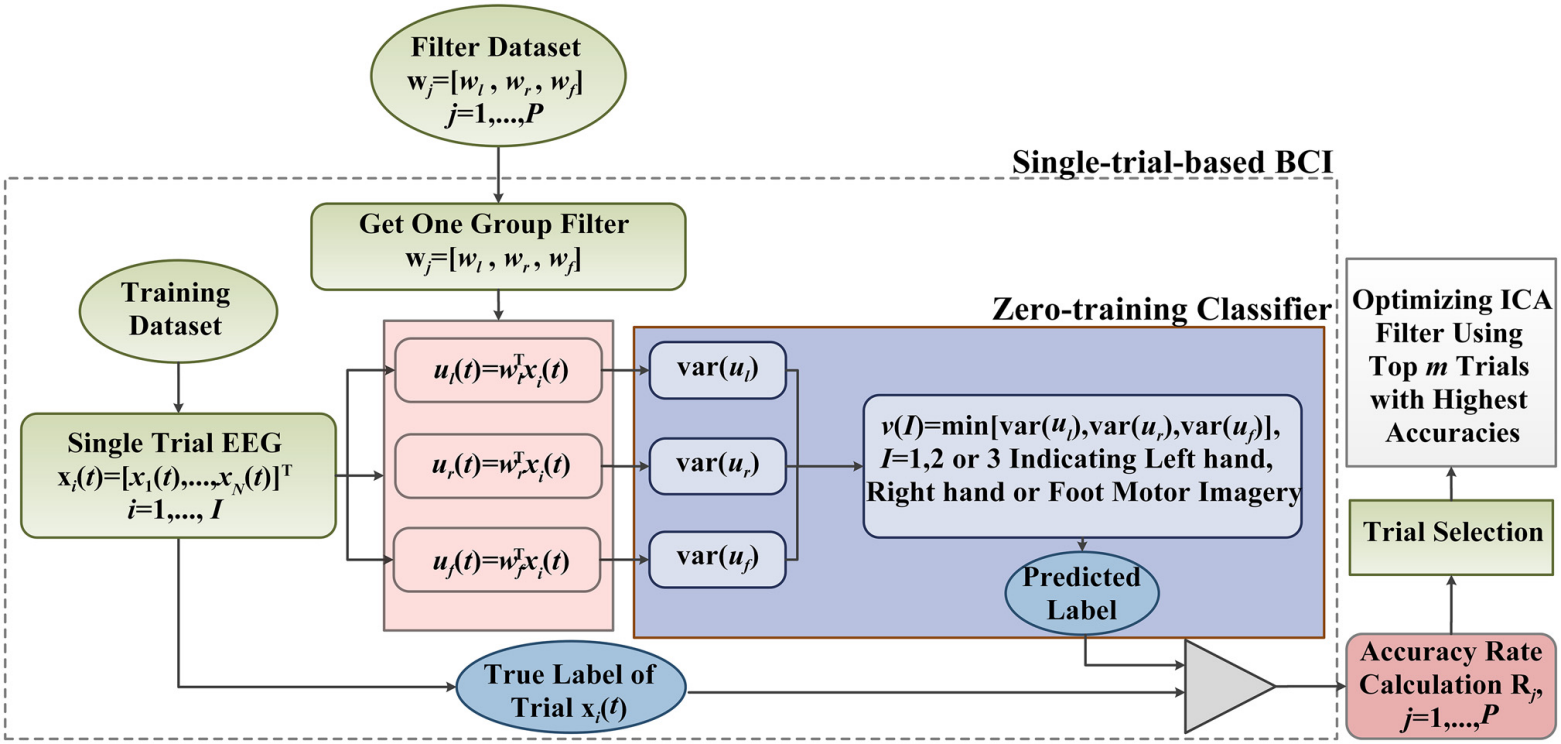
The schematic drawing of the second-round data selection algorithm.

In the second-round trial selection, the detection filters: {w_*j*_ = [*w*_*l*_, *w*_*f*_, *w*_*r*_], *j =* 1,…,*P*} apply to BCI for single-trial EEG classification in training dataset. As shown in Fig 8, each of MRICs detection filters is individually applied to the algorithm frame to construct a BCI testing system. This “single-trial-based BCI” is referred to as st-BCI, because the spatial filters [*w*_*l*_, *w*_*f*_, *w*_*r*_] in st-BCI are calculated on a single trial EEG. Thus, we can get *P* st-BCIs based on filter datasets {w_*j*_ = [*w*_*l*_, *w*_*f*_, *w*_*r*_], *j =* 1,…,*P*}.

In Fig 8, each spatial filter **w**_*j*_ is employed by st-BCI to derive the MRICs from EEG trials **x**_*i*_ (*i =* 1,…,*I*) in the training dataset. For one single trial, three MRICs ***u***_*l*_(*t*), ***u***_*r*_(*t*) and ***u***_*f*_(*t*) corresponding to left hand, right hand and foot movement imagination are obtained by spatial filters **w**_*j*_ = [***w***_*l*_, ***w***_*f*_, ***w***_*r*_], namely:

$$u_{l}(t)=w_{l}^{Τ}x_{i};\;u_{r}(t)=w_{r}^{Τ}x_{i};\;u_{f}(t)=w_{f}^{Τ}x_{i}$$ (7)

Their variances in a range of 0.5–5 s (see Fig 1) are used to construct the feature vectors for a zero-training classifier. The classification rule is just based on the well-known ERD phenomena [18–19] induced by limb movement imagination. The classification rule can be described as follows:

$$V=min[var(u_{l}),var(u_{r}),var(u_{f})]$$ (8)

$$trial\;\begin{array}{c} x_{i} \end{array}\in \{\begin{array}{lll} class\;1 & left\;hand\;imagination & if\;\begin{array}{r} V=var(u_{r}) \end{array}\\ class\;2 & right\;hand\;imagination & if\;\begin{array}{c} V=var(u_{l}) \end{array}\\ class\;3 & foot\;imagination & if\;\begin{array}{c} V=var(u_{f}) \end{array} \end{array}$$ (9)

The classifier output predicts the label of movement imagination for each trial in training dataset, and gives the classification accuracy *R*_*j*_ associated with a spatial filter **w**_*j*_ = [***w***_*l*_, ***w***_*f*_, ***w***_*r*_]. Since each spatial filter **w**_*j*_ = [***w***_*l*_, ***w***_*f*_, ***w***_*r*_] was calculated on one corresponding good trial, it is reasonable to adopt classification accuracies *R*_*j*_, *j* = 1,…,*P* as the reference for further trial selection. So, the rule for the second-round trial selection is that if the classification accuracy *R*_*j*_ is below a specific threshold, it will also be treated as a bad trial.

**Fig 9:**
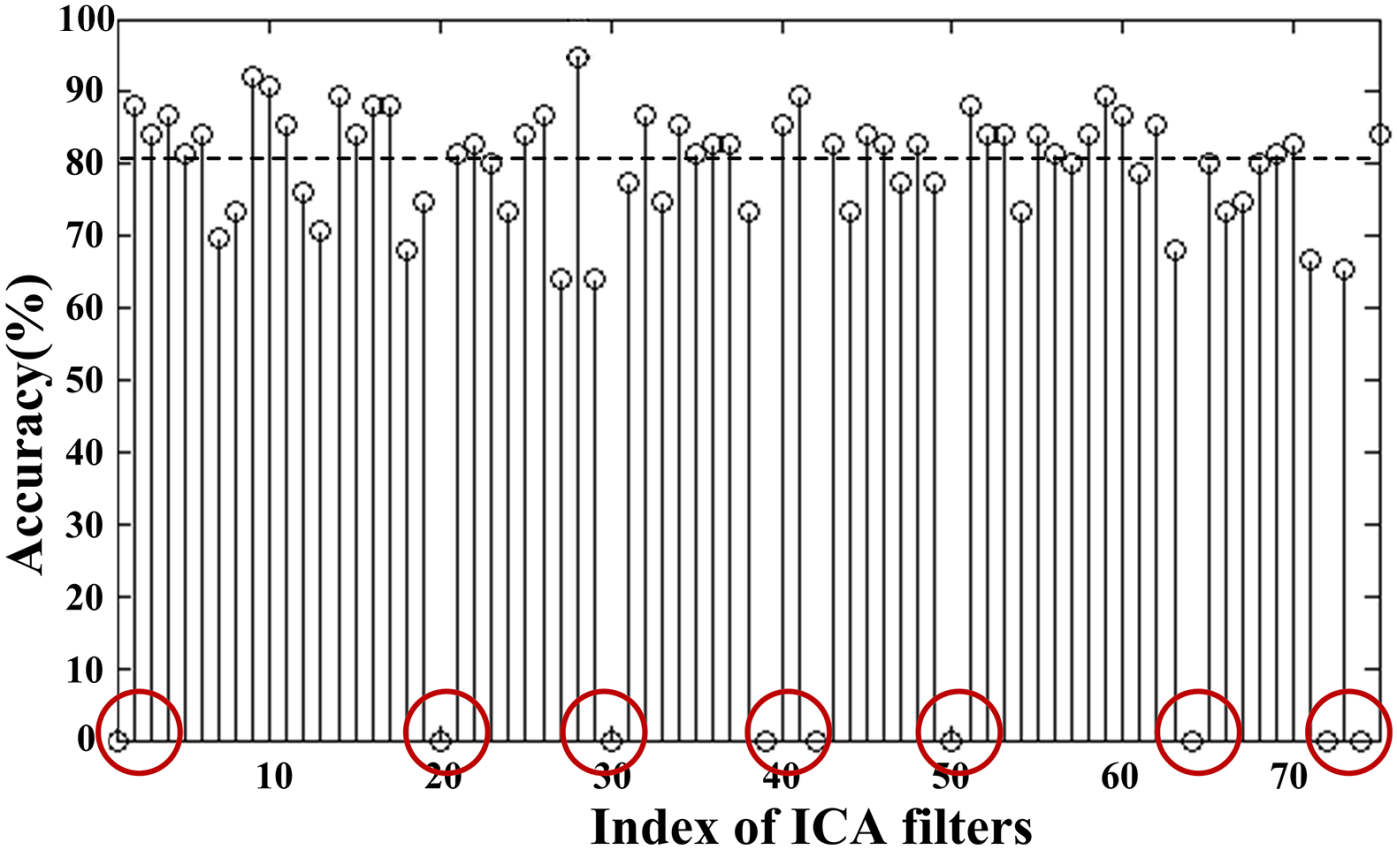
The accuracies of the st-BCIs calculated on one run training dataset. The average accuracy was 80.6% (indicated by the horizontal dashed line), and the accuracies marked by red circles correspond to bad trials after first-round selection.

The selection procedure can be illustrated in Fig 8, in which a training set of 75 trials (*I* = 75) is evaluated by the two-round trial selection algorithms depicted in Figs 6 and 8. The accuracy rate *R*_*j*_ shown in Fig 9 indicates that there are 9 trials of zero accuracy rates (marked by red circles), which are considered to be "bad trials" at the first-round selection. The remaining 66 trials (*P* = 66) and corresponding spatial filters are then selected for the second-round analysis yielding 66 st-BCI and non-zero accuracies (see Fig 9). The average indicated by the horizontal dashed line is 80.6%, and there are 35 accuracy rates of st-BCI smaller than the average. The trials corresponding to lower-accuracies in Fig 9 may be corrupted by artifacts and do not provide enough accurate information for ICA learning.

#### 3.2.3 Final MRICs detection filters design

In this part, the reserved trials in training data from the two-round selection are sorted descendingly according to st-BCI recognition accuracies. The “top *m* trials” with the highest accuracies are selected and concatenated to recalculate the ICA filters. For easy description, we named the proposed algorithm “ICA-T” in this paper. In this study, *m* was set to be 10 for 8 or 9-channel EEG analysis. We employed another ICA filter calculation method to evaluate the performance of this optimization algorithm. This ICA method named “ICA-S” in this study is characterized by calculating the ICA over a sliding window of 10 trial length at a step of one trial length. Furthermore, the accuracies obtained by the two types of ICA-based MIBCIs were compared with that obtained by CSP-based MIBCI [35].

## Results

### 4.1 Self-testing

24 runs of motor imagery EEG data were used in this study, with 6 runs data for each subject. According to the “ICA-T” algorithm, the optimized MRICs filters are based on “top 10 trials” for each run, and one self-testing accuracy could be obtained after applying the optimized filters to the same run. The “ICA-S” algorithm sequentially selects 10 trials to calculate MRICs spatial filters. It thus amount to 65 groups of MRICs filters for each run data. The final self-testing accuracy is calculated by averaging the 65 testing results. For the CSP algorithm, 60 trials (80% of 75 trials in one run) were selected randomly to train CSP spatial filters, which were then applied to test all 75 trials. This process was repeated 30 times and the self-testing accuracy was calculated by averaging 30 testing results.

**Fig 10:**
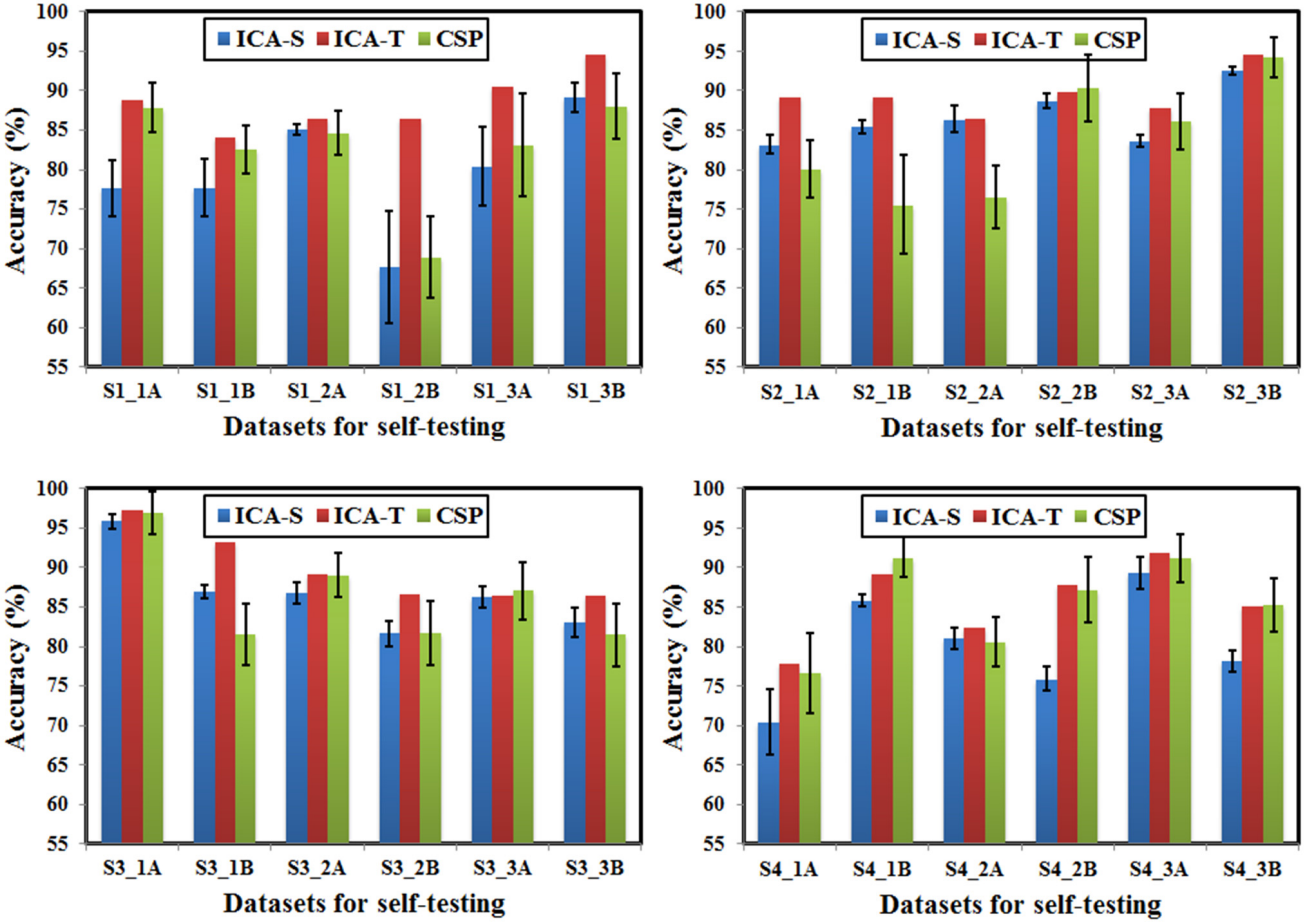
The intra-run self-testing classification accuracies of three-class motor imagery, using the “ICA-T”, “ICA-S” and CSP algorithm respectively. Bar graphs are shown with standard deviation.

The comparisons of self-testing accuracies of three algorithms are illustrated in Fig 10. It can be seen that the “ICA-T” algorithm outperforms “ICA-S” algorithm for all 24 runs data, the self-testing accuracies are significantly improved, and the maximum increase reaches 18.86% (S1_2B). Compared with the “ICA-S” algorithm, the CSP algorithm produces higher accuracies in most cases (16 runs out of all 24 runs), possibly because of the utility of more information for CSP (more training data and pre-known labels). However, “ICA-T” algorithm outperforms CSP algorithm in 20 runs with the maximum increase of 13.58% (S2_1B), and in the leftover 4 runs, CSP algorithm is a little better than the “ICA-T” algorithm, with the maximum increase of 2.15% (S4_1B). These results demonstrate the effectiveness of trial-selection-based ICA optimization algorithm.

**Fig 11:**
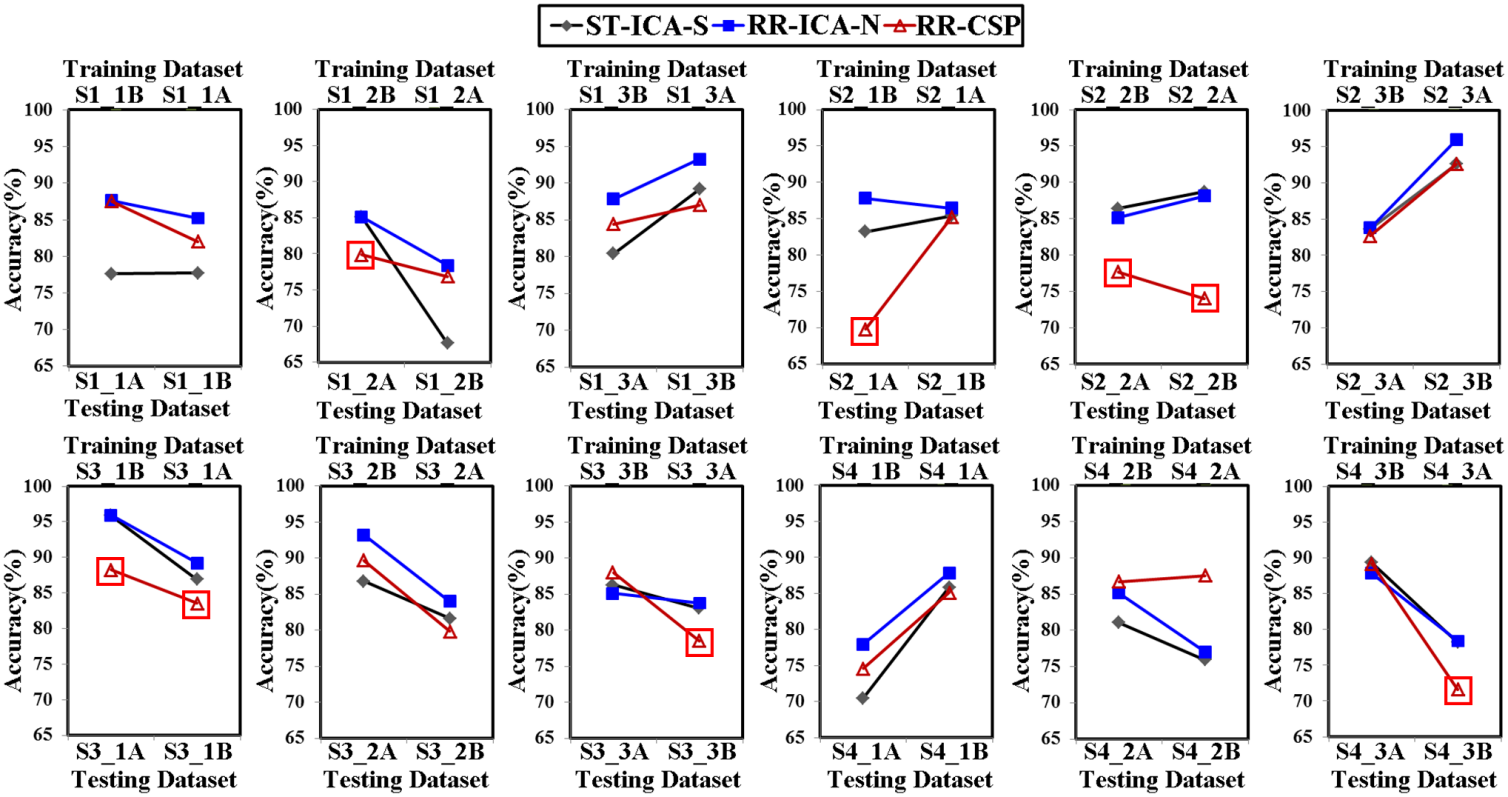
The accuracies of run-to-run transfer using “ICA-T” and CSP algorithm respectively (represented by “RR-ICA-T” and “RR-CSP” respectively), compared with the intra-run self-testing accuracies calculated with “ICA-S” algorithm (represented by “ST-ICA-S”).

In order to evaluate the robustness of above mentioned spatial filtering algorithm, the optimized spatial filters calculated in one run using “ICA-T”/CSP algorithm were applied to test the other runs measured on the same day. Note that all 75 trials in one run were used to train CSP spatial filters. Moreover, the accuracies of inter-run testing were compared with the self-testing accuracies of “ICA-S” algorithm. As shown in Fig 11, the “ICA-T” algorithm presents good performance in the inter-run validation for all 24 runs data, the 20 accuracies of inter-run validation are still higher than the self-testing accuracies for the “ICA-S” algorithm with the maximum increase of 10.75% (S1_2A: training set, S1_2B: testing set), and the accuracies of remaining 4 runs are slightly lower with the maximum decrease of 1.5% (S3_3B: training set, S3_3A: testing set). While for CSP algorithm, the accuracy of inter-run validation drops sharply in most cases (see the rectangle markers in Fig 11), and the highest decline amounted to 14.66% (S2_2A: training set, S2_2B: testing set).

### 4.2 Session-to-session transfer

The session-to-session transfer [36–39] is often performed by utilizing spatial filters from previous sessions and sharing the parameters with the new session. In this section, for each subject, each run dataset from one session was selected to calculate spatial filters using “ICA-T” and CSP algorithm respectively to test 4 runs datasets from another two sessions. So, 24 groups of accuracies of session-to-session transfer were obtained, and they were compared with the self-testing accuracies of testing datasets using the “ICA-S” algorithm.

**Fig 12:**
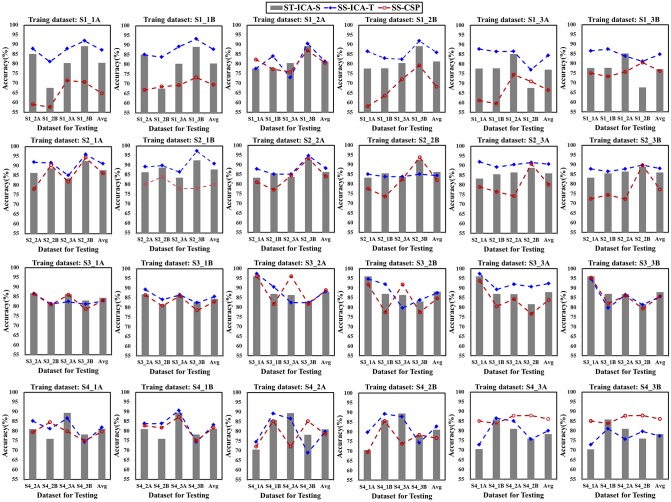
The classification accuracies of session-to-session transfer using “ICA-T” and CSP algorithm (represented by “SS-ICA-T” and “SS-CSP” respectively), compared with the results of intra-run self-testing accuracies calculated with “ICA-S” algorithm (represented by “ST-ICA-S”).

As indicated in Fig 12, the accuracy of inter-session test by the “ICA-T” algorithm is higher than that of the self-test accuracy by the “ICA-S” algorithm for most of the cases (67 out of 96 cases). This is especially true for the data with high artifact interference (for instance, S1_2B, S1_3A, S1_3B, S4_1A), where the maximum increase is 13.46% (S1_3B to S1_2B transfer). For the data with less satisfactory self-test accuracy, the “ICA-T” spatial filter also exhibits good performance in the session-to-session transfer. For example, the self-test accuracy of S1_2B is only 67.62%, while a higher accuracy is obtained for all the cases of the session-to-session transfer (S1_2B to S1_1A, S1_1B, S1_3A and S1_3B) using “ICA-T” algorithm, the highest one improves 8.9% (S1_2B to S1_1A transfer). The CSP results present much fluctuating performances and exhibit apparently lower accuracy than the self-test accuracy for most cases (70 out of 96).

## Discussions

### 5.1 The influence of burst artifacts on ICA calculation

**Fig 13:**
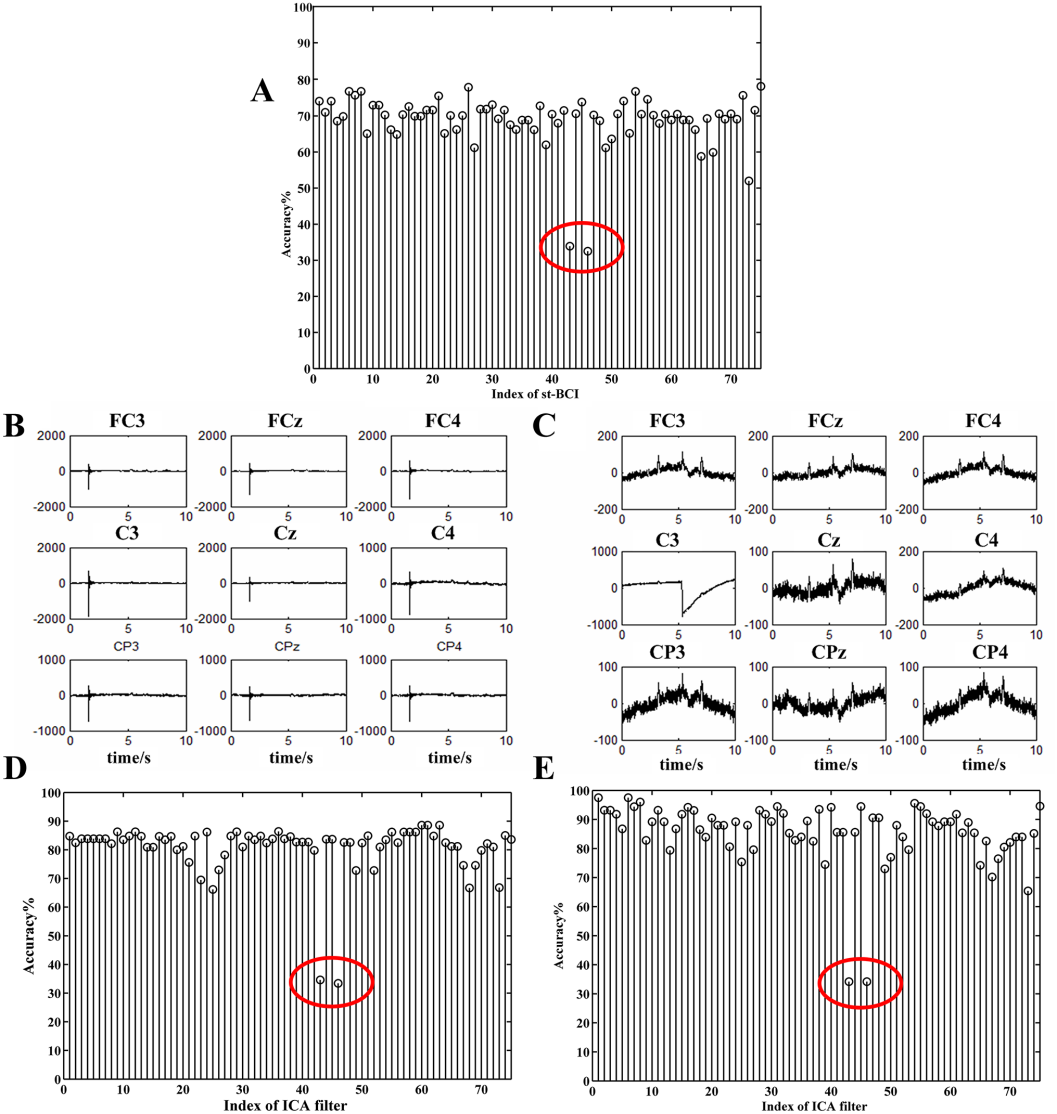
**(A) The self-testing accuracies of S1_a dataset in st-BCI.** The red-circle marked accuracies (R_43_, R_46_) were apparently lower. **(B) The nine-channel EEG signals of the 43rd trial. (C) The nine-channel EEG signals of the 46th trial. (D)(E) The accuracies of session-to-session transfer with S1_a as training dataset and S1_b and S1_c as testing datasets respectively.**

In this section, we will illustrate the degradation caused by burst of artifacts. We chose three out of the six runs EEG datasets from subject S1 (named by S1_a, S1_b and S1_c) to demonstrate this phenomenon. S1_a were used to train the ICA_BCI, and the S1_b and S1_c were used for testing. After two-round trial selection algorithm to S1_a, the recognition accuracy of st-BCIs is shown in Fig 13A. It shows that no trials are labeled bad in the first-round selection, while two accuracies (*R*_43_ and *R*_46_, marked by red circles) are apparently smaller than the others. We can see that the corresponding trials were seriously contaminated by burst interferences (see Fig 13B and 13C). A very low accuracy is also appeared in the same index (see Fig 13D and 13E) when the st-BCI is applied to test S1_b and S1_c.

**Fig 14:**
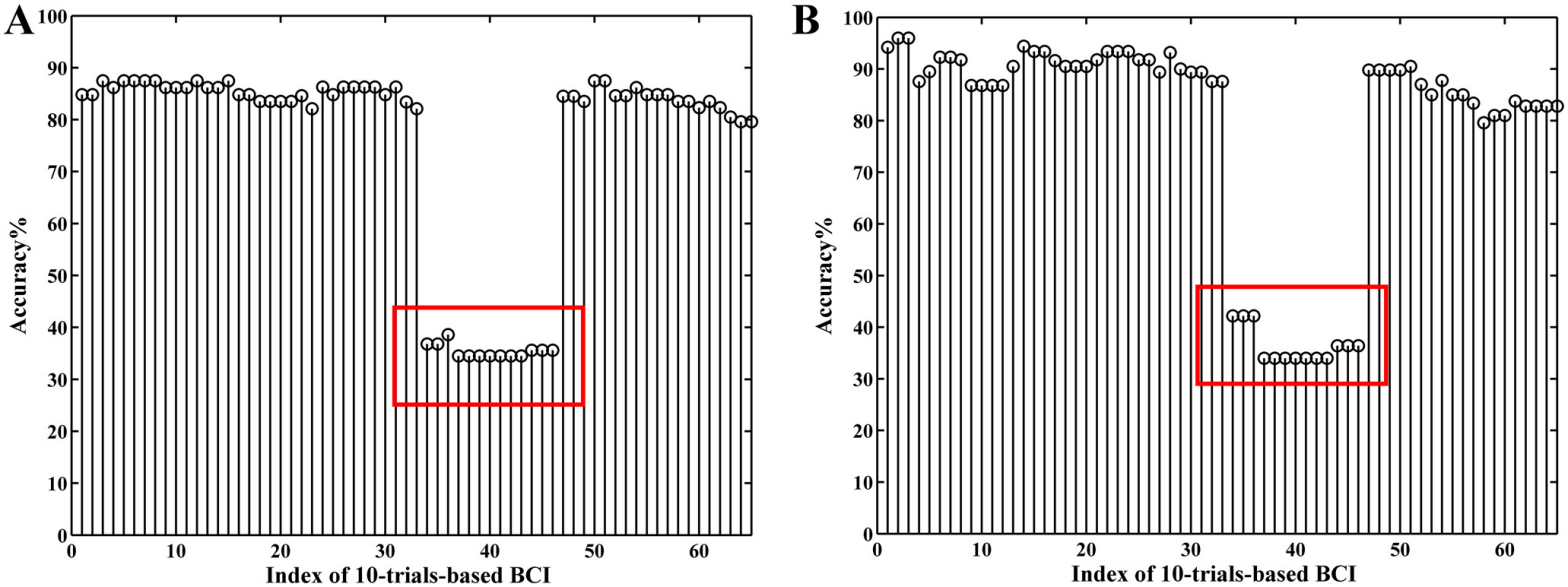
The accuracies of session-to-session transfer using 10 trials sequentially selected from S1_a to train ICA filters which were applied to test the S1_b (A) and S1_c (B) respectively.

More results are given in Fig 14 to illustrate the influence of artifact trials. In this example, we selected 10 sequential trials from continuous EEG samples in S1_a to get 10-trial-based ICA filters instead of single-trial-based ones in the second-round trial selection. The adjacent EEG sequences for the ICA filter calculation were overlapped with 9 trials. We can have 65 10-trial-based ICA-BCIs based on S1_a. When applying them to test S1_b and S1_c, the accuracies from the 34th to 46th BCIs are apparently lower than the others (Fig 14). This is because the corresponding ICA filters (34th to 46th) were derived from trials that contain at least one of the two artifact trials (***x***43, ***x***46).

### 5.2 Accuracy-matrix: a visualization tool for detailed artifact analysis

We now introduce a method called accuracy-matrix (AM) to visualize the location of the artifact segments within a trial. The AM consists of the classification accuracies of BCIs designed by consecutively overlapping EEG segments from each trial in one EEG dataset. Assuming that *T*_*t*_ denotes the length of a trial and *T*_*s*_ the length of a EEG segment, *T*_*o*_ the length of overlap of neighbor segments, then the number of EEG segments obtained from one trial is:

$$M=round(\frac{T_{t}-T_{o}}{T_{s}-T_{o}})$$ (10)

where *round*(·) is a function that round a number to the nearest integer. For a dataset containing *L* EEG trials, we can get *M*×*L* EEG segments. Each segment is used to construct a BCI with the proposed method (see Fig 6), and the final classification accuracies were put into an AM of size *M*×*L* for subsequent analysis.

**Fig 15:**
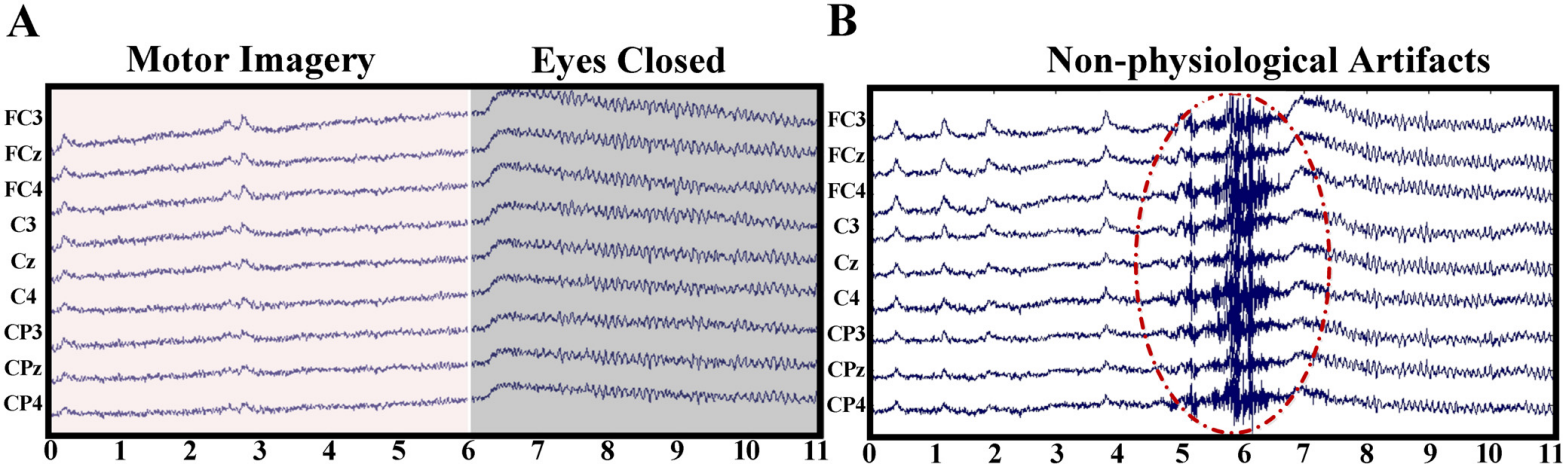
Two examples of raw EEG data with different types of artifacts. (A) Raw 9-channel EEG signals of one trial in S1_4 dataset during motor-imagery and eyes-closed time segments respectively. (B) Raw 9-channel EEG signals of one trial in S1_5 dataset with obvious non-physiological artifacts.

We can get four AMs (two self-test, and two session-to-session transfers) for datasets S1_4 and S1_5. The EEG acquisition protocol for S1_4 and S1_5 is similar to that shown in Fig 1 except that: (1) the total time duration *T*_*t*_ of single trial is 11 s; (2) at the end of motor imagination process (at 6 s), the subject was asked to keep the eyes closed till the warning tone of next trial. Each dataset contains 75 single trials, 25 trials for each class of motor imagery. Fig 15 shows the waveforms of two trials selected from S1_4 and S1_5 respectively, which presents evident physiological artifacts (such as EOG, alpha wave) and burst non-physiological interferences appearing at the 9-channel EEG signals during motor imagery and eye closing periods. Thus, the EEG segments used for ICA filter calculation are likely contaminated by different types of artifacts to some extent.

In the following testing experiment, the duration of the segment *T*_*s*_ was 5 s, and the overlap with the neighbor segments was 4.5 s. It thus produced 13 segments per trial for ICA filters calculation according to eq (10). We can obtain 975 (75×13) ICA filters for a dataset of 75 trials, which produces the classification accuracies {*R*(*i*, *j*), *i* = 1,…,13; *j* = 1,…,75} to construct an AM of size 13×75.

**Fig 16:**
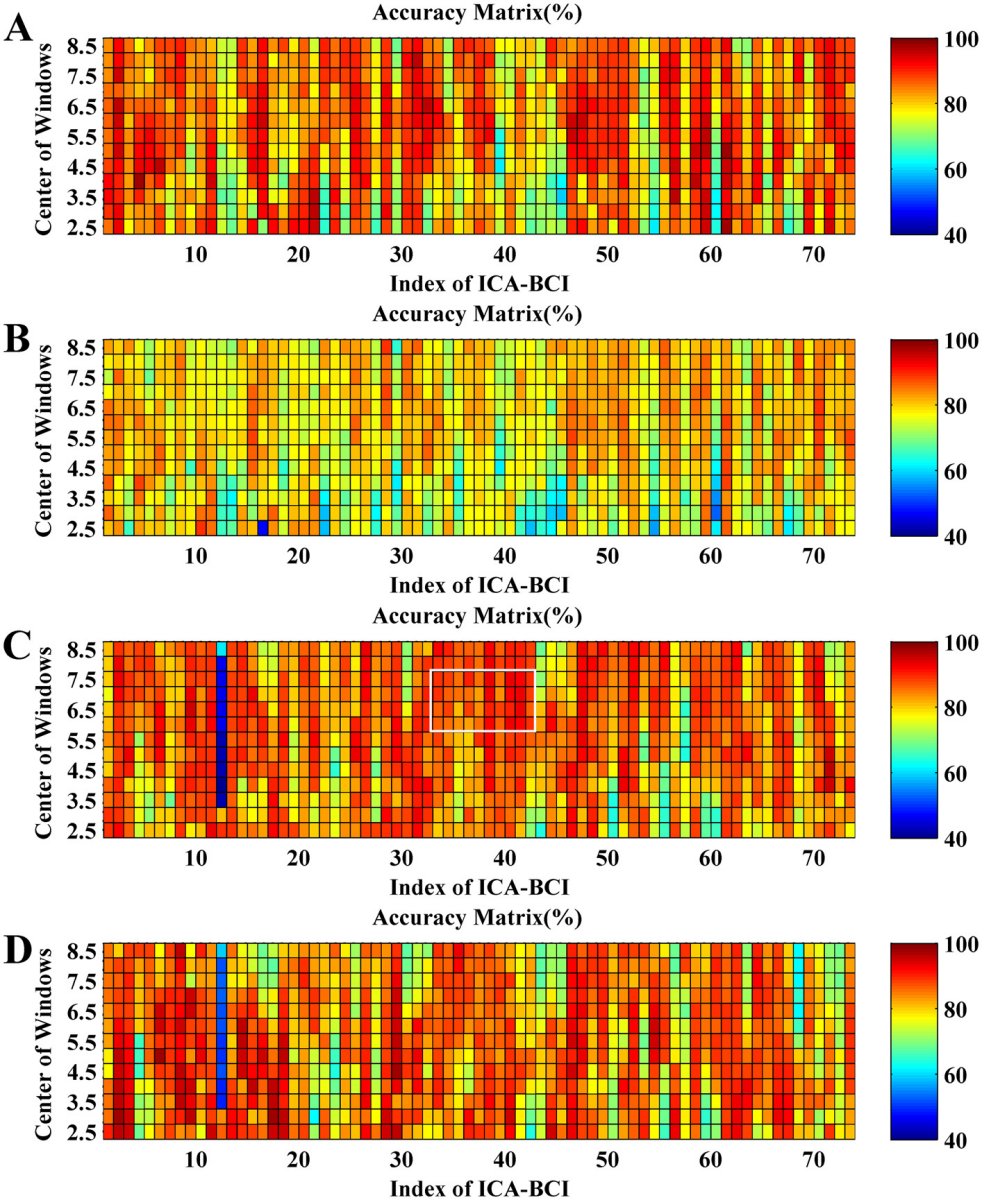
The accuracy matrixes of self-testing and session-to-session transfer using different time segments in each single trial to calculate ICA filters. (A) The self-testing of S1_4; (B) S1_4 to S1_5 transfer; (C) The self-testing of S1_5; (D) S1_5 to S1_4 transfer.

**Fig 17:**
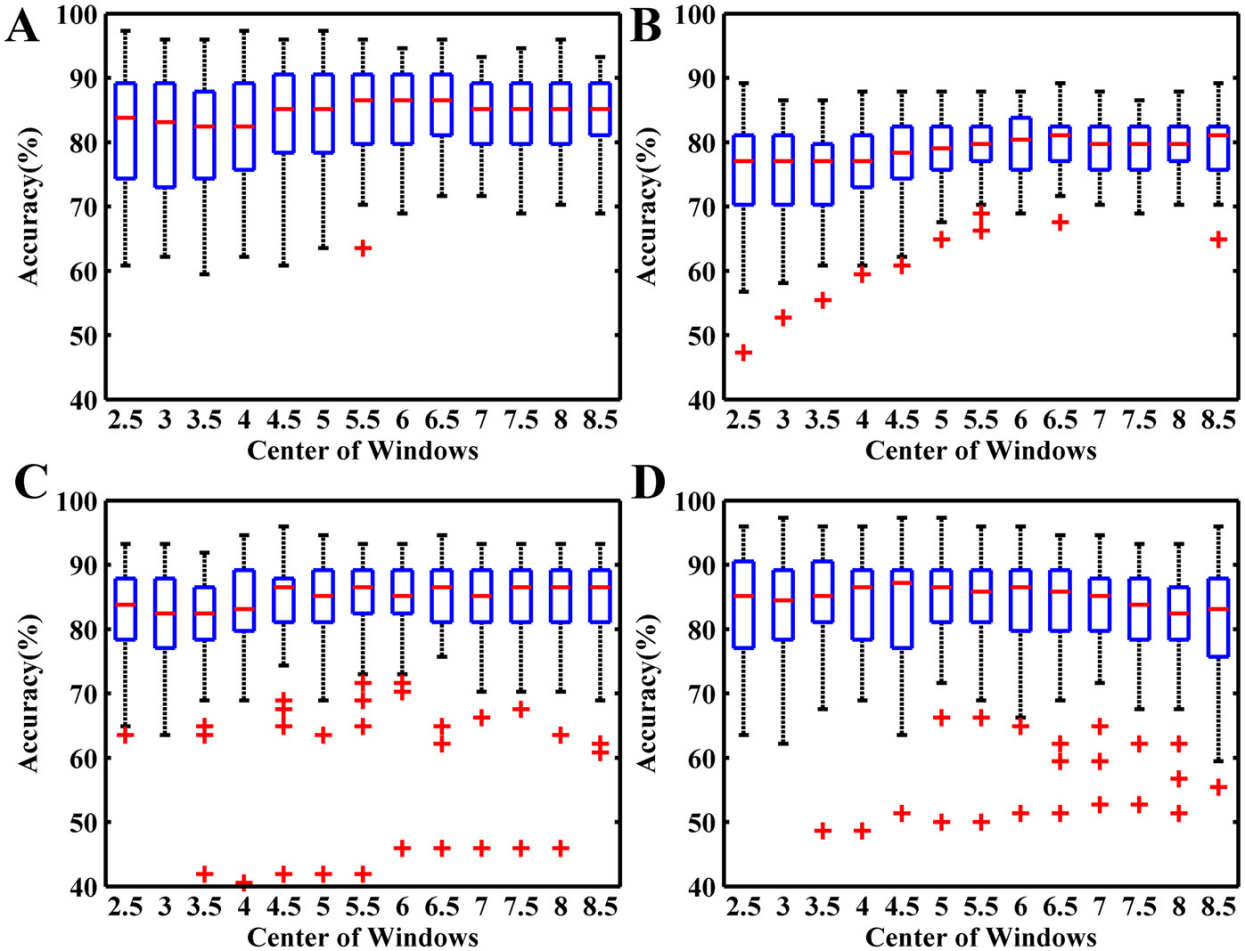
The boxplots of accuracies of self-testing and session-to-session transfer in Fig 16 with different sliding windows centres. (A) The self-testing of S1_4; (B) S1_4 to S1_5 transfer; (C) The self-testing of S1_5; (D) S1_5 to S1_4 transfer.

Fig 16 shows the AMs of self-testing and session-to-session transfer for S1_4 and S1_5. One small square at the *i*th row and *j*th column represents the accuracy of a BCI based on a different ICA filter derived from the *i*th EEG segment (5 s length) of the *j*th trial. So, each row of AM is corresponding to the same time location in different trials and each column related to the same single trial with different time segments. Fig 17 shows the corresponding boxplot of each row of AM in Fig 16.

By observing the four AMs and the corresponding statistical values in Fig 17, we cannot observe any apparent relations between accuracies and the time location of EEG segments. For instance, the average accuracy of {*R*(*i*,*j*), *i* = 3,^…^,6 *j* = 33,^…^,42} is 91.0% as indicated by the white box in Fig 16C, while the EEG segments corresponding to the region are located at the beginning of eyes-closed period, which contains high-amplitude EOGs and alpha waves induced by eyes closing (see Fig 15A). These EEG segments are usually excluded from the calculation of the spatial filter [36,40]. However, according to the accuracy in the AMs, the performance of ICA based MIBCI is unlikely affected by these physiological artifacts. Fig 16C and 16D show the results of self-testing of S1_5 and the session-to-session transfer of S1_5 (training) and S1_4 (testing). It appears that, most entries of the 12th columns of the two matrices (corresponding to the 12th EEG trials in S1_5) are extremely lower compared with others. The reason can be found in Fig 15B which shows the signal of the 12th trial of S1_5 containing apparent burst noise between 5 and 7 s.

By inspecting all EEG trials with the AM method, we found that the performance of ICA filters might be degraded by burst interferences induced by occasional moving of body, cable and electrodes connection loosing, etc., rather than by the normal physiological artifacts.

## Conclusion

In this study, we investigated the influences of different artifacts on ICA-based MIBCI. Since the unpredictable non-physiological artifacts would induce performance degradation and instability of ICA algorithm, we proposed a fully automated method to detect the artifact trials without using any predefined templates of the typical artifacts. The results demonstrate that ICA spatial filters optimized with high-quality trials can significantly improve the performance of ICA-based MIBCIs, and is superior to the well-known CSP-based MIBCI, especially in the session-to-session transfer.

Compared with the commonly used high-density channels [14,22,40–44] in ICA-based BCI researches, this study employed much fewer channels (only 8 or 9 channels) and shorter-time EEG samples (less than 100 s) to get the ICA spatial filter, which can greatly shorten the preparation time for data acquisition and calibration. By combining the optimized ICA filter and zero-training classifier, we present a potential to build a testing platform of MIBCI techniques with low computation complexity. The proposed method enables us to carry out further analysis related to BCI testing and optimizing, such as AMs-based training sample selection, which endows the ICA with broader applicability in MIBCI. The electrode distribution is a major factor affecting the performance of the proposed ICA-based MIBCIs, so future work will focus on investigating the ICA-based automatic channel selection algorithm to replace the manual selection.
